# Investigating the effects of NOTCH3 Asian‐specific mutant on blood‐brain‐barrier using patient iPSC‐derived cell types

**DOI:** 10.1002/alz70855_106310

**Published:** 2025-12-24

**Authors:** Jolene Wei Ling Lee, Adeline Su Lyn Ng, Eng‐King Tan, Li Zeng

**Affiliations:** ^1^ Duke‐NUS Medical School, Singapore, Singapore; ^2^ National Neuroscience Institute, Singapore, Singapore

## Abstract

**Background:**

Cerebral autosomal dominant arteriopathy with subcortical infarcts and leukoencephalopathy (CADASIL), is the genetic form of vascular dementia leading to vascular impairments and cognitive decline. CADASIL is caused by mutations in the NOTCH3 gene which encodes a single‐pass transmembrane receptor. The NOTCH3 mutant (MT) is reported to be the most common risk variant in Asia with almost 1% of community controls in Singaporean Chinese are carriers. Despite clinical observations, this variant has yet to be listed as a “pathogenic” variant due to the lack of functional studies done. Several reports indicated that Blood‐Brain‐Barrier (BBB) dysfunction can be the driving mechanism for synaptic dysfunction in CADASIL. Our work aims to model NOTCH3 MT pathogenesis and study its effects on vascular function with patient‐derived induced pluripotent stem cells (iPSC)‐derived BBB cell types.

**Method:**

Patient peripheral blood monocnuclear cells (PBMC) obtained were reprogrammed into iPSC. These MT iPSC were corrected via CRISPR‐Cas9 to serve as an isogenic control (Corrected). After which, MT and corrected iPSCs were differentiated into endothelial cells (EC), pericytes (PC) and astrocytes (AC) with previously described protocols. Functional assays such as angiogenesis, migration and trans‐endothelial electrical resistance (TEER) assays were carried out. Subsequently, these cell types were incorporated into a 3D *in vitro* platform, forming BBB‐like microtissues.

**Result:**

In comparison with corrected cell types, expression of NOTCH3 in MT cell types were varied, with no significant differences in ECs and decreased expression in ACs and PCs. MT ECs and PCs also displayed opposite expression trend of tight junction marker, occludin, suggesting a compensatory mechanism. In addition, MT ECs displayed decreased angiogenesis, migration and barrier tightness. BBB‐like microtissues were able to spontaneously form perfusable vascular structures after 7 days in culture which can serve as a promising tool to study vascular function and properties.

**Conclusion:**

Our findings show that the effects of NOTCH3 MT is multi‐faceted, affecting multiple cell types, calling for the need to study them in a heterogenous model. Ultimately, unravelling effects of vascular deregulation in CADASIL will contribute to understanding its disease pathophysiology.

